# Iron(III) Fluorinated Porphyrins: Greener Chemistry from Synthesis to Oxidative Catalysis Reactions

**DOI:** 10.3390/molecules21040481

**Published:** 2016-04-12

**Authors:** Susana L. H. Rebelo, André M. N. Silva, Craig J. Medforth, Cristina Freire

**Affiliations:** 1 REQUIMTE/LAQV, Departamento de Química e Bioquímica, Faculdade de Ciências, Universidade do Porto, Rua do Campo Alegre, 4169-007 Porto, Portugal; acfreire@fc.up.pt; 2 REQUIMTE/UCIBIO, Departamento de Química e Bioquímica, Faculdade de Ciências, Universidade do Porto, Rua do Campo Alegre, 4169-007 Porto, Portugal; andre.silva@fc.up.pt (A.M.N.S.); craig.medforth@fc.up.pt (C.J.M.)

**Keywords:** iron(III)porphyrin synthesis, microwave, green oxidation, catalysis, aromatics

## Abstract

Iron(III) fluorinated porphyrins play a central role in the biomimetics of heme enzymes and enable cleaner routes to the oxidation of organic compounds. The present work reports significant improvements in the eco-compatibility of the synthesis of 5,10,15,20-*tetrakis*-pentafluorophenylporphyrin (H_2_TPFPP) and the corresponding iron complex [Fe(TPFPP)Cl], and the use of [Fe(TPFPP)Cl] as an oxidation catalyst in green conditions. The preparations of H_2_TPFPP and [Fe(TPFPP)Cl] typically use toxic solvents and can be made significantly greener and simpler using microwave heating and optimization of the reaction conditions. In the optimized procedure it was possible to eliminate nitrobenzene from the porphyrin synthesis and replace DMF by acetonitrile in the metalation reaction, concomitant with a significant reduction of reaction time and simplification of the purification procedure. The Fe(III)porphyrin is then tested as catalyst in the selective oxidation of aromatics at room temperature using a green oxidant (hydrogen peroxide) and green solvent (ethanol). Efficient epoxidation of indene and selective oxidation of 3,5-dimethylphenol and naphthalene to the corresponding quinones is observed.

## 1. Introduction

Fluorinated porphyrins have been attracting considerable attention as components of photonic materials and in biological applications, such as imaging or photodynamic therapy, owing to their versatile photophysics, electron withdrawing properties and resistance to oxidative degradation [1,2,3,4,5].

On the other hand, iron and manganese porphyrins have been successfully tested as biomimetics of heme enzymes, including cytochrome P450 monooxygenases [6,7], peroxidases [8] or catalases [9,10]. The use of synthetic metaloporphyrin models has brought important insights into the mechanisms and intermediates of the biological reactions [11]. In addition, biomimetic models are advantageous since they are readily available and much easier to handle than the bio-catalytic systems, with halogenated metaloporphyrins being among the first used, most efficient and robust catalytic systems [12,13].

**Figure 1:**
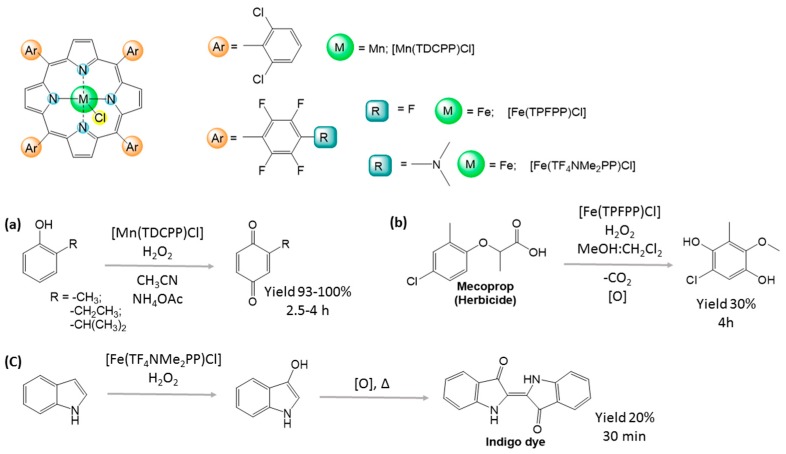
Oxidations of aromatic compounds in the presence of Mn(III) or Fe(III)porphyrins: (**a**) oxidation of alkylphenols; (**b**) oxidation of the herbicide mecoprop; (**c**) production of indigo dye from indene oxidation.

In the last few decades, studies of catalytic oxidations using metaloporphyrins as P450 models have led to new oxidation routes and new compounds that would otherwise be difficult to obtain, such as multi-epoxides of polycyclic aromatic hydrocarbons [14,15]. Furthermore, metaloporphyrin catalysis afforded novel synthetic methods for important chemicals with improved eco-compatibility [16,17], using green oxidants such as hydrogen peroxide [18,19] and mild conditions [20]. For example, manganese porphyrins were used in the selective and mild oxidation of alkylphenols to *p*-quinones using H_2_O_2_ at room temperature. This procedure was efficient in acetonitrile using ammonium acetate as a co-catalyst (Figure 1a) [21]. Subsequently, iron fluorporphyrin systems were found to show higher efficiency than manganese analogous for hydroxylation of aromatic rings in solvents such as methanol:dichloromethane, namely during the oxidation of the herbicide mecoprop to the *p*-hydroquinone derivative (Figure 1b) [22]. More recently, it was hypothesized that the high efficiency observed in the biomimetic production of indigo dye, the color of blue jeans, was due to the hydroxylation of indole in the presence of an Fe(III)porphyrin system (Figure 1c) [16].

One of the major drawbacks of these methodologies is the hazardous solvents used for the porphyrin synthesis and metalation reactions and the long heating times for metalation reactions, which reduce the eco-sustainability and feasibility of these procedures. For example, the commonly used porphyrin 5,10,15,20-*tetrakis*-pentafluorophenyl porphyrin (H_2_TPFPP) that is the topic of this paper is typically prepared by condensation of pyrrole and pentafluorobenzaldehyde in a refluxing mixture of acetic acid and nitrobenzene followed by a complex purification procedure [23,24]. The Fe(III) metalation procedure to produce [Fe(TPFPP)X] is generally based on the Adler method [25], which consists of refluxing H_2_TPFPP with a large excess of the ferrous salt in *N*,*N*-dimethylformamide (DMF) and pyridine for 48 h. Furthermore, in the case of H_2_TPFPP, this process results in the substitution of the *p*-fluorine atoms by nucleophilic attack of dimethylamine, a product of DMF decomposition [26]. The partial or complete substitution of the *p*-fluorine atoms by 4-dimethylamine groups effectively prevents functionalization by nucleophilic substitution, which is often an important synthetic pathway for the modification of H_2_TPFPP [27] or its metal complexes, e.g., for anchoring them to solid supports and for the preparation of hybrid materials for catalysis and photonic applications [28,29]. Although other solvents have been used to replace DMF, all the methodologies were characterized by long reaction times, the use of high temperatures and harmful or corrosive solvents.

Some microwave procedures have been reported to be efficient for the synthesis of metaloporphyrins [30,31,32]. However, green procedures for halogenated porphyrins synthesis and their metalation using Fe have not been described. Such methods are potentially of significant interest given the environmentally hazardous procedures currently employed in the preparation of fluorinated metaloporphyrins.

With the aim of demonstrating a more eco-compatible process from catalyst synthesis to oxidative catalysis, the present work reports the optimization of the synthesis and metalation of H_2_TPFPP using microwave heating. The study involves a systematic evaluation of the reaction solvent, time, temperature and reagent concentrations. Electrospray ionization Orbitrap mass spectrometry (ESI-Orbitrap-MS) was used to characterize the iron(III) complexes [33] and evaluate nucleophilic substitution of the *p*-fluorines by dimethylamine. The catalytic activity of the iron(III) complex was then evaluated in the oxidation of indene, 3,5-dimethylphenol and naphthalene by a green oxidant (H_2_O_2_) in a green solvent (ethanol) without other additives.

## 2. Results and Discussion

### 2.1. Optimization of the Microwave Synthesis of H_2_TPFPP

A study of microwave synthesis was performed in order to achieve a faster and greener route to the preparation of H_2_TPFPP. The optimization of the procedure started from previous reports of metaloporphyrin synthesis [23,30] and involved a systematic evaluation of the reaction conditions.

**Figure 2:**
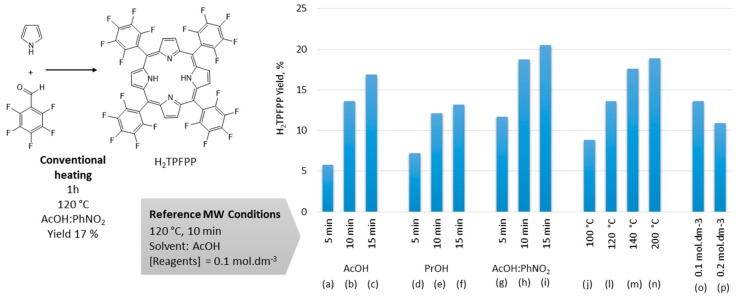
Synthesis of H_2_TPFPP in conventional heating conditions and screening of the microwave (MW) reaction conditions. The reference conditions were used and (**a**–**c**) 5, 10, 15 min reaction times; (**d**–**f**) propionic acid as solvent with 5, 10, 15 min as reaction times; (**g**–**i**) acetic acid:nitrobenzene mixture as solvent with 5, 10, 15 min as reaction times; (**j**–**n**) different reaction temperatures; (**o**–**p**) different concentrations of reagents. The yields were determined by UV-Vis spectroscopy.

In the method of Gonsalves *et al.* [23,24], nitrobenzene was used simultaneously as a co-solvent and oxidant (to decrease the formation ofreduced macrocycles, which brings additional difficulties to the chromatographic separation). Is this synthesis, conventional heating (at reflux) of a mixture of acetic acid and nitrobenzene (2:1) containing pentafluorobenzaldehyde and pyrrole (0.1 mol·dm^−3^) for 1 h yielded 17% H_2_TPFPP as quantified by UV-Vis analysis (Figure 2). However, under these conditions the porphyrin did not precipitate directly from the reaction mixture, as reported for other *meso*-tetraphenylporphyrins, and it is necessary to separate the H_2_TPFPP from an abundance of polymeric material by column chromatography. After purification the isolated yield of H_2_TPFPP was ~11%.

A reference set of microwave conditions was first developed (Figure 2): 0.1 mol·dm^−3^ reagents heated in acetic acid (AcOH) at 120 °C for 10 min [30]. In order to compare the efficiency of the modified reactions, yields were evaluated by UV-Vis analysis of the reaction mixture. Different solvents were studied, acetic acid (AcOH), propionic acid (PrOH, allowing a higher reflux temperature) and a mixture of acetic acid and nitrobenzene (AcOH:PhNO_2_) and various reaction times were tested (5, 10 and 15 min) (Figure 2, conditions a–i).

**Figure 3:**
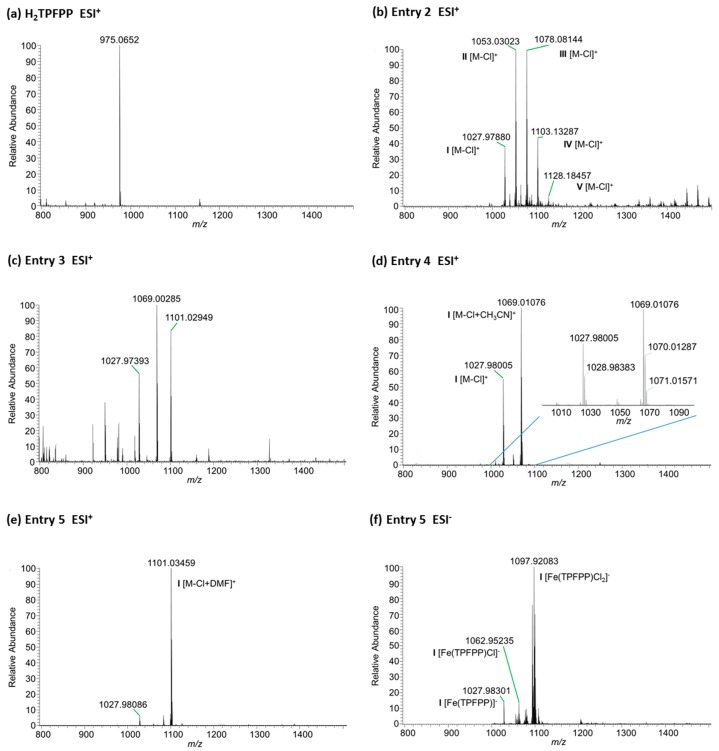
High Resolution ESI mass spectra analysis of H_2_TPFPP (**a**) and of iron complexes obtained under the experimental conditions described in Table 1, Entry 2 (**b**); Entry 3 (**c**); Entry 4 (**d**); Entry 5 (**e**,**f**).

In all solvents the highest yields were obtained for 15 min reactions. Higher yields were obtained in AcOH:PhNO_2_ relative to other solvents, although a good yield was also achieved for the reaction in pure AcOH. The worst result after 15 min was obtained for the reaction in PrOH. This may be due to the higher pka of PrOH (4.87) relatively to AcOH (4.75) leading to lower efficiency in the acid catalyzed formation of porphyrinogen. Furthermore, the reaction in AcOH (conditions c) shows a slightly higher amount of probably a reduced macrocycle form; as detected by the presence of the electronic band near 730 nm (the UV-Vis spectra can be seen in Supplementary Materials, Figure S1a). However, it was observed that the H_2_TPFPP precipitated when the AcOH reactions were set aside for 72 h. After filtration, washing with hexane and recrystallization from dichromethane:*n*-hexane mixture, pure H_2_TPFPP was recovered as verified by TLC analysis and mass spectrometry (Figure 3a). This solved the purification problems associated with chlorin formation and established AcOH as the most eco-compatible solvent.

The effect of different reaction temperatures on the reaction in AcOH for 10 min were then investigated (conditions j, l, m and n). The yield increases with temperature but the difference between 140 °C (17.6%) and 200 °C (18.9%) was small. Doubling the concentration of the reagents had a negative effect on the yield (conditions o and p), although the decrease in yield was small enough that the amount of porphyrin produced was considerably higher at a concentration of 0.2 mol·dm^−3^.

These results demonstrate a significant reduction of reaction time and increased eco-compatibility for microwave synthesis using acetic acid and 0.1 mol·dm^−3^ reagent concentrations, with H_2_TPFPP being isolated in 13% yield by precipitation from the reaction mixture after 72 h. This greener route compares very favorably with the 17% yield obtained by conventional heating in AcOH:PhNO_2_ for much longer periods, and the subsequent laborious chromatographic separation leading to a final yield of ~11%. Further evidence of the advantage of microwave heating was obtained by comparing the isolated yields for two reactions performed in acetic acid at 120 °C for 1 h using conventional heating and for 10 min in microwave heating, 5% and 14%, respectively (Figure S1b).

### 2.2. Optimization of the Iron Insertion Reaction Using Microwave Heating Conditions

**Figure 4:**
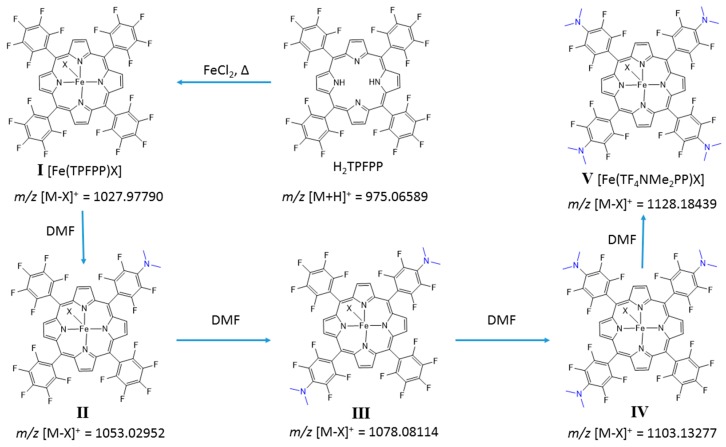
Complexes of [Fe(TPFPP)X] obtained during metalation reactions by heating DMF and pyridine mixtures for long reaction periods.

The application of Adler’s procedure [25] to the preparation of Fe(III) and Mn(III) complexes of H_2_TPFPP often leads to uncontrolled substitution of the *p*-fluorine atoms of the pentafluorophenyl rings due to nucleophilic attack by dimethylamine arising from decomposition of DMF [26]. The DMF decomposition probably occurs by a thermal degradation mechanism, [34] although DMF can also be hydrolyzed by water present in the solvent. The resulting Fe(III)porphyrin complexes are depicted in Figure 4. Compound **I** is the desired unsubstituted chloro [5,10,15,20-*tetrakis*(2,3,4,5,6-pentafluorophenyl)porphyrinate] iron(III) complex. Compound **V** is the chloro [5,10,15,20-*tetrakis*(4-dimethylamino-2,3,5,6-tetrafluorophenyl)porphyrinate] iron(III) where all of the *p*-fluorine groups have been replaced by dimethylamine groups. Further nucleophilic substitution of the *p*-phenyl positions of compound **V** is hampered and consequently its derivatization is limited [29].

Compound **V** is the main complex obtained using an experimental procedure based on the Adler method. This consists of refluxing a solution of H_2_TPFPP in DMF and pyridine (10:1 *v*/*v*) using conventional heating and adding 10 molar equivalents of FeCl_2_. The reaction is monitored by TLC and addition of the same amounts of iron salt and pyridine continued until full conversion of the H_2_TPFPP was achieved (Table 1, Entry 1). The resulting product was analyzed by mass spectrometry (Supplementary Materials, Figure S2). The most intense peak *m*/*z* 1128.2 corresponded to the ion [M − Cl]^+^ from the tetra-substituted derivative [Fe(TF_4_NMe_2_PP)Cl] (**V**).

The optimization studies started by comparing the iron insertion procedure at a lower reaction temperature (120 °C) using conventional and microwave heating (Table 1, Entries 2 and 3, respectively). In the microwave heating reaction, pyridine was eliminated from the reaction mixture. The reaction using conventional heating (Entry 2), lasted 48 h and the ratio of iron salt: H_2_TPFPP was 200. For the microwave reaction (Entry 3), the final reaction time and the iron salt to H_2_TPFPP where decreased to 24 h and 100, respectively.

The resulting compounds were analyzed by high resolution mass spectrometry with electrospray ionization in the positive mode (ESI^+^-MS). The data is shown in Figure 3. The spectrum of product obtained using conditions on Entry 2 (Figure 3b), shows the molecular ions [M − Cl]^+^ of all five possible metaloporphyrins (**I**–**V**). However, the most intense peaks are seen for the mono- and di-substituted derivatives **II** (*m*/*z* 1053.03023) and **III** (*m*/*z* 1078.08144).

On the other hand, the first microwave procedure that is described in Entry 3 led not only to the reduction of reaction time and amount of iron salt but also avoided the substitution of *p*-fluorine atoms by dimethyl amino groups (Figure 3c). The species observed in the mass spectrum corresponded to complex **I** [M − X]^+^ as adducts with DMF (*m*/*z* 1101.02949), or with CH_3_CN (*m*/*z* 1069.00285) or the complex without solvent (*m*/*z* 1027.97393). The peak [M − X + CH_3_CN]^+^ is also observed in other analysis of this compound when the solvent used to dissolve the sample is CH_3_CN [33].

Subsequent studies led to a significant improvement of the reaction conditions. The reaction described on Entry 4 of Table 1 considered the use of acetonitrile as the reaction solvent replacing DMF and pyridine. Furthermore, the initial reaction mixture was stirred at room temperature for 1 h before microwave heating at 120 °C for 30 min. After monitoring the reaction by TLC, the procedure was repeated with addition of a second aliquot of iron salt (10 molar equivalents relative to H_2_TPFPP), followed by the stirring at room temperature and microwave heating procedures. After this second addition, the metalation of H_2_TPFPP was complete. These conditions led to a significant reduction of the reaction time (finished in 3 h, considering the agitation and heating periods) and amount of iron salt required to achieve complete metalation (Entry 4). The effect of stirring the reaction mixture prior to heating is probably due to the fact that coordination of iron to the porphyrin occurs more readily in the ferrous state, and upon heating of the reaction mixture the ferric state is easily produced and the metalation rate is significantly reduced.

The mass spectrum in the positive mode (ESI^+^) of the compound resulting from the reaction conditions in Entry 4 (Figure 3d) shows two significant peaks, the peak [M − X]^+^ from complex **I** [Fe(TPFPP)X] (*m*/*z* 1027.98005) and its adduct with acetonitrile (*m*/*z* 1069.01076). Nucleophilic substitution is also avoided under these conditions and derivative **I** is obtained selectively.

The microwave synthesis was performed at 160 °C in DMF (Entry 5) in an attempt to determine if it was possible to achieve complete substitution of the *p*-fluorine atoms by dimethylamine groups to produce compound **V**. However, only the non-substituted derivative **I** was obtained in the form of its adduct with DMF (Figure 3e). The dimethylamine is probably only formed when there is prolonged heating of DMF.

Finally, the same compound was also analyzed using ESI mass spectrometry in the negative mode (ESI^−^), and the spectrum is shown in Figure 3f. The most intense peak corresponds to the ion [Fe(TPFPP)Cl_2_]^−^, which is complex **I** with two chlorine atoms as the axial ligands (Figure 3f).

The methodology developed for the synthesis [Fe(TPFPP)Cl] embodies a significant improvement in the preparation of the iron complexes of fluorinated porphyrins. As far as we are aware, these are the first studies to use microwave heating for the synthesis of [Fe(TPFPP)Cl].

### 2.3. Green Oxidation of Aromatic Compounds Using [Fe(TPFPP)Cl]

**Figure 5:**
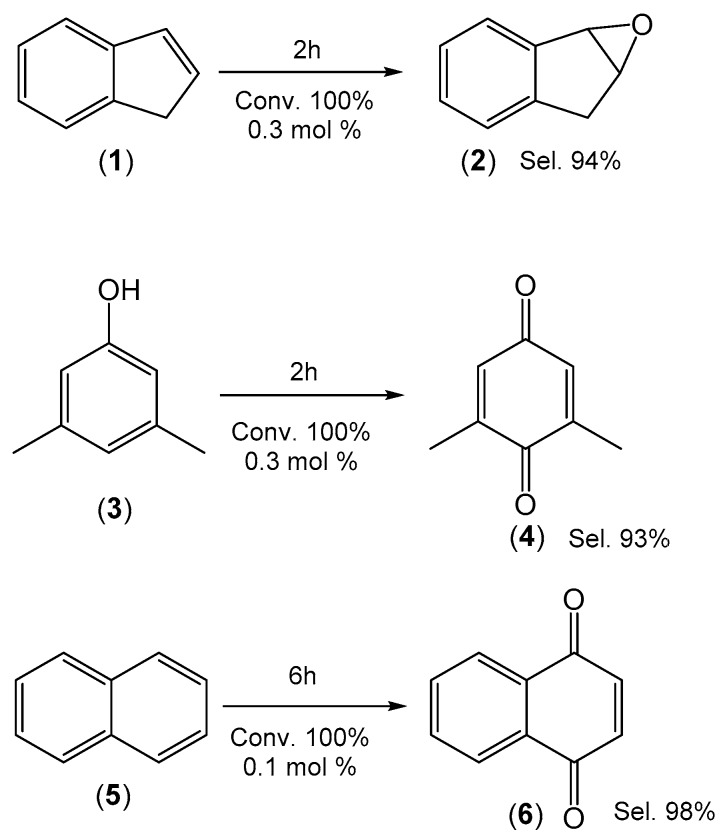
Catalytic oxidation of aromatic compounds in the system [Fe(TPFPP)Cl]/EtOH/H_2_O_2_.

The [Fe(TPFPP)Cl] prepared using the reaction described in Entry 4 of Table 1 was then tested in the oxidation of aromatic compounds using green conditions. The oxidation of indene (1), 3,5-dimethylphenol (3) and naphthalene (5) was studied using hydrogen peroxide as a green oxidant at room temperature. Ethanol was used as the solvent system without the presence of other additives (Figure 5).

Using 0.3 mol % of catalyst, indene (1) is oxidized to indene oxide (2) with full conversion and 94% selectivity in a reaction time of 2 h (Figure S3). Epoxidation at the 1,2-position, preferential to the hydroxylation reaction, can be related to the alkene character present at this position [14,15]. The other products all show higher retention times than indene oxide, with their amounts increasing for higher reaction temperatures. These compounds probably result from epoxide ring opening reactions. The predominance of the epoxidation pathway indicates a mechanism proceeding through a high valent iron porphyrin species rather than the generation and attack of hydroxyl radicals [11,16].

Using the same conditions, 3,5-dimethylphenol (3) was completely and selectively oxidized to 3,5-dimethyl-1,4-benzoquinone (4) (Figures S4–S6). No traces of the 1,2-benzoquinone are observed in the ^1^H-NMR and ^13^C APT NMR spectra of the total reaction mixtures (Figures S5 and S6), which only show the signals expected for the more symmetrical 1,4-benzoquinone. The oxidation of naphthalene (5) using the same conditions but 1 mol % of catalyst afforded 1,4-naphthoquinone (6) in quantitative yield after a reaction time of 6 h (Figures S7 and S8). The selectivity for the 1,4-naphthoquinone (6) increases in comparison with reactions catalyzed by manganese porphyrins [35]. The hydroxylation reaction can also be explained by the action of the high valent ironporphyrin species, as has been reported both for metaloporphyrin models [11,16,22] and P-450 mutants [36]. Control experiments involving oxidation of the three substrates (1, 3, and 5) using the above conditions but in the absence of catalyst were performed and the conversions were always below 5%. For indene oxidation the GC chromatograms of the oxidation reactions in the presence and absence of catalyst are shown in the supporting material (Figure S9).

## 3. Materials and Methods 

All the reagents and solvents were used as received without further purification. Pyrrole (98%), indene (98%), 3,5-dimethylphenol (>99%) and naphthalene (99%) were purchased from Aldrich (St. Louis, MO, USA), and pentafluorobenzaldehyde (98%) was purchased from Acros Organics-Fisher Scientific (Porto Salvo, Portugal). *N*,*N*-dimethylformamide (DMF), ethanol (EtOH), acetonitrile, acetic acid (AcOH), propionic acid (PrOH), nitrobenzene (PhNO_2_), chloroform, *n*-hexane and FeCl_2_.4H_2_0 were of *p.a.* grade. Hydrogen peroxide was 30% *w*/*w* (Pedrogen, Aldrich).

### 3.1. Physical Measurements

UV-visible absorption spectra were recorded on a UV-3600 Spectrophotometer (Shimadzu Corporation, Kyoto, Japan). High-resolution electrospray ionization mass spectra (ESI-MS) were obtained using a LTQ-Orbitrap XL mass spectrometer (Thermo Scientific, Bremen, Germany), externally calibrated with a standard kit provided by the manufacturer. Samples were dissolved in acetonitrile and directly infused into the electrospray ion source at 10 µL·min^−1^ utilizing the syringe pump in the mass spectrometer. The spectrometer was operated in the positive or negative ionization mode with the capillary voltage set to +3.1 kV or −1.7 kV, respectively, sheath gas flow to 6 and the temperature of the ion transfer capillary to 275 °C.

NMR spectra and attached proton test (APT) experiments of the compounds and total reaction mixtures were recorded using a Advance III spectrometer (Brucker, Billerica, MA, USA) operating at a frequency of 400 MHz and 100 MHz for ^1^H and ^13^C experiments, respectively, with sample temperatures of 22 °C.

Analyses using gas chromatography with flame ionization detection (GC-FID) were performed using a CP-3380 gas chromatograph (Varian, Palo Alto, CA, USA) with helium as the carrier gas and a fused silica Varian Chrompack capillary column CP-Sil 8 CB Low Bleed/MS (30 m × 0.25 mm i.d.; 0.25 µm film thickness). The temperature program was: 70 °C (1 min), 20 °C·min^−1^, 200 °C (5 min). The injector temperature was 200 °C and the detector temperature 250 °C.

Analyses using gas chromatography-mass spectrometry (GC/MS) were performed using a Finnigan Trace GC/MS Thermo Quest (CE instruments, Lancashire, UK) and helium as the carrier gas (35 cm/s). The capillary column and the temperature program were the same as described for the GC-FID analyses.

### 3.2. Preparation of Metaloporphyrin Catalysts

Microwave syntheses were carried out using a Discover LabMate reactor (CEM Corporation, Matthews, NC, USA) with a temperature control system equipped with a reaction vessel of 10 mL. The reaction mixture was constantly irradiated in a closed system, at the desired temperature, using up to 300W of power.

For the studies on the synthesis of H_2_TPFPP, 6 mL of the reaction solvent were introduced into the reaction vessel, or when a solvent mixture was used, acetic acid (4 mL) and nitrobenzene (2 mL) were added to the reaction vessel. The reagents were added to the microwave reactor immediately before the reactions were heated. For the reactions performed at reagent concentrations of 0.1 mol.dm^−3^, pyrrole (6.0 × 10^−4^ mol; 41.6 μL) and pentafluorobenzaldehyde (6.0 × 10^−4^ mol; 74.1 μL) were added to the reaction vessel and the mixture agitated using a magnetic stirrer for the chosen temperature and time (Figure 2). The completed reactions were promptly analyzed using UV-Vis spectroscopy using solutions diluted with chloroform.

In the reactions performed in acetic acid, after 72 h a porphyrin precipitate was observed. The reaction mixture was filtered, the precipitate washed with *n*-hexane and recrystallized from dichloromethane:*n*-hexane.

For the studies of the preparation of the iron complex [Fe(TPFPP)Cl], the reaction vessel was charged with 5 mL of the reaction solvent and 50 mg of H_2_TPFPP and the vessel irradiated using the conditions described in Table 1. For the reactions described in Entries 4 and 5, the initial reaction mixture was agitated for 1 h outside of the microwave apparatus prior to irradiation.

The iron insertion reactions were evaluated by thin layer chromatography (TLC) using silica plates with chloroform as eluent to determine if metalation was complete. The solvent was then evaporated and the reaction mixture dissolved in chloroform and washed several times with deionized water. After evaporation of chloroform, the metaloporphyrin was dissolved in dichloromethane and reprecipitated by the addition of *n*-hexane. The precipitate was filtered and washed with *n*-hexane.

### 3.3. Catalysis Experiments

In a typical experiment, the substrate (0.3 mmol) and the metaloporphyrin (1 or 3 μmol) were dissolved in 2 mL of ethanol and stirred at 22 °C. Aqueous hydrogen peroxide (30% *w/w*) diluted in ethanol (1:10) was added to the reaction mixture at a rate of 0.6 mL/h through a syringe pump. Control experiments in the absence of catalyst were also performed. The indene reactions were monitored by GC-FID using decane as an internal standard and 1,2-indene oxide produced by a reference method [19] for product identification. The naphthalene reactions were monitored by thin layer chromatography (silica gel 60 DGF254) using CH_2_Cl_2_ as the eluent. At the reaction end, the 1,4-naphthoquinone (**6**) was isolated by TLC and its structure confirmed by ^1^H- and ^13^C-NMR and GC-MS analysis with electronic impact ionization (EI). The oxidation reactions of 3,5-dimethylphenol (**3**) were analyzed by removing the solvent using a rotavapor keeping the water bath at room temperature. The total reaction mixtures were analyzed then by MS-ESI and NMR (^1^H and APT).

### 3.4. Analytical Data for Compounds

(Metalo)porphyrins

H_2_TPFPP: MS (ESI^+^) *m*/*z*: 975.0652 [M + H]^+^, calculated: 975.0659, Δm: 0.7 ppm.

Compound **I**: MS (ESI^+^) *m*/*z*:. 1027.97880 [Fe(TPFPP)]^+^, calculated: 1027.97790, Δm: 0.9 ppm.

Compound **II**: MS (ESI^+^) *m*/*z*: 1053.03023 [M − Cl]^+^, calculated: 1053.02952, Δm: 0.7 ppm

Compound **III**: MS (ESI^+^) *m*/*z*: 1078.08144 [M − Cl]^+^, calculated: 1078.08114, Δm: 0.3 ppm.

Compound **IV**: MS (ESI^+^) *m*/*z*: 1103.13287 [M − Cl]^+^, calculated: 1103.13277, Δm: 0.1 ppm.

Compound **V**: MS (ESI^+^) *m*/*z*: 1128.18457 [M − Cl]^+^, calculated: 1128.18439, Δm: 0.2 ppm.

*Indene oxide* (**2**) MS (ESI^+^) *m*/*z*: 133.06441 [M + H]^+^, calculated: 133.06479, Δm: 2.9 ppm.

*3,5-dimethyl-1,4-benzoquinone* (**4**) ^1^H-NMR (DMSO-*d*_6_, 400 MHz) δ (ppm): 1.98 (*s*, 6H, –CH_3_), 6.66 (*s*, 2H, H-2,6). ^13^C-NMR (DMSO-*d*_6_, 100 MHz, APT) δ (ppm): 16.2 (–CH_3_), 133.4 (C-2,6), 146.1 (C3,5), 188.4 (C-1,4). MS (ESI^+^) *m*/*z*: 137.05918 [M + H]^+^, calculated: 137.05971, Δm: 3.9 ppm.

*1,4-naphthoquinone* (**6**) ^1^H-NMR (CDCl_3_, 400 MHz) δ (ppm): 6.98 (*s*, 2H; H-2,3), 7.74-7.79 (*m*; 2H; H-6,7), 8.07-8.11 (*m*; 2H; H-5,8). ^13^C-NMR (CDCl_3_, 100 MHz, APT): 126.4 (C-5,8), 132.0 (C-4a,5a), 134.0 (C-6,7), 138.7 (C-2,3), 185.1 (C-1,4). EM (EI) *m*/*z* (%): 158 (M^+•^; 100); 130 (30).

## 4. Conclusions

The syntheses of H_2_TPFPP and [Fe(TPFPP)Cl] have been optimized using microwave methods to allow the production of these important fluorinated porphyrins in a more eco-sustainable manner, and the use of the prepared [Fe(TPFPP)Cl] has been investigated in green catalytic oxidation reactions. The synthesis of H_2_TPFPP in 13% isolated yield was achieved by microwave heating of 0.1 M pyrrole and pentafluorobenzaldehyde in acetic acid at 120 °C for 15 min. The H_2_TPFPP then precipitates from the reaction mixture after 72 h and is isolated by filtration, washing and recrystallization. The metalation of H_2_TPFPP to form the corresponding iron complex [Fe(TPFPP)Cl] was achieved by stirring H_2_TPFPP for 1 h with 10 equivalents of FeCl_2_ in acetonitrile at room temperature, followed by microwave heating of the reaction mixture for 30 min at 120 °C and repeating once the addition of FeCl_2_ procedure and heating. The [Fe(TPFPP)Cl] is isolated in quantitative yield, whereas the standard metal insertion procedure using DMF produces varying amounts of porphyrins where the *para*-fluorine groups have undergone nucleophilic substitution by dimethylamine produced by decomposition of the solvent. The oxidation of indene (1), 3,5-dimethylphenol (3) and naphthalene (5) at room temperature using a green oxidant (hydrogen peroxide) and solvent (ethanol) was then investigated. High selectivity (>90%) was observed for the formation of indene oxide (2), 3,5-dimethyl-1,4-benzoquinone (4) and 1,4-naphthoquinone (6) using 0.1 and 0.3 mol % of catalyst and reaction times of 2–6 h.

## Figures and Tables

**Table 1:** Reaction conditions for the metalation reactions using conventional and microwave heating.

Entry	Heating	Solvent	Temperature	Time	FeCl_2_:H_2_TPFPP ^a^
1	conventional	DMF, Py	155 °C	48 h	200
2	conventional	DMF, Py	120 °C	48 h	200
3	microwave	DMF	120 °C	24 h	100
4	microwave	CH_3_CN	120 °C ^b^	3 h ^b^	20
5	microwave	DMF	160 °C ^b^	3 h ^b^	20

^a^ Molar ratio of “iron salt:H_2_TPFPP” at the end of reaction; ^b^ After addition of each FeCl_2_ aliquot, the reaction was stirred for 1 h at room temperature before heating (the reaction times shown refer to the total time, agitation + heating time).

## Supplementary Materials

Supplementary materials can be accessed at: http://www.mdpi.com/1420-3049/21/4/481/s1.
