# Supplementary material for: Iron(III) Fluorinated Porphyrins: Greener Chemistry from Synthesis to Oxidative Catalysis Reactions
Source: Molecules. 2016 Apr 12;21(4):481. doi: 10.3390/molecules21040481 (PMC6274165; doi:10.3390/molecules21040481)
Supplement: Supplementary file 1 [file molecules-21-00481-s001.pdf]

Supplementary Material: Iron(III) Fluorinated Porphyrins: Greener Chemistry from Synthesis to Oxidative Catalysis Reactions

Susana L. H. Rebelo, André M. N. Silva, Craig J. Medforth and Cristina Freire

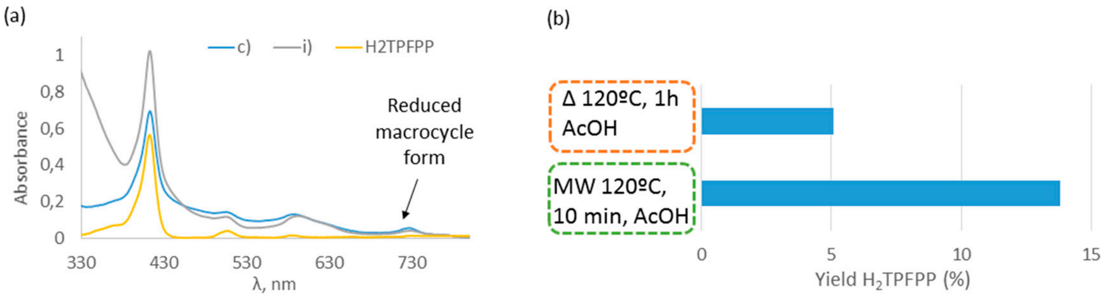

Figure S1. (**a**) UV-Vis spectra of the reactions performed in microwave heating under conditions c and i of Figure 2; (**b**) comparison of the isolated yields of H_2TPFPP under conventional (upper) and microwave (lower) heating using acetic acid as the reaction solvent and 0.1 mol dm^{-3} reactant concentrations.

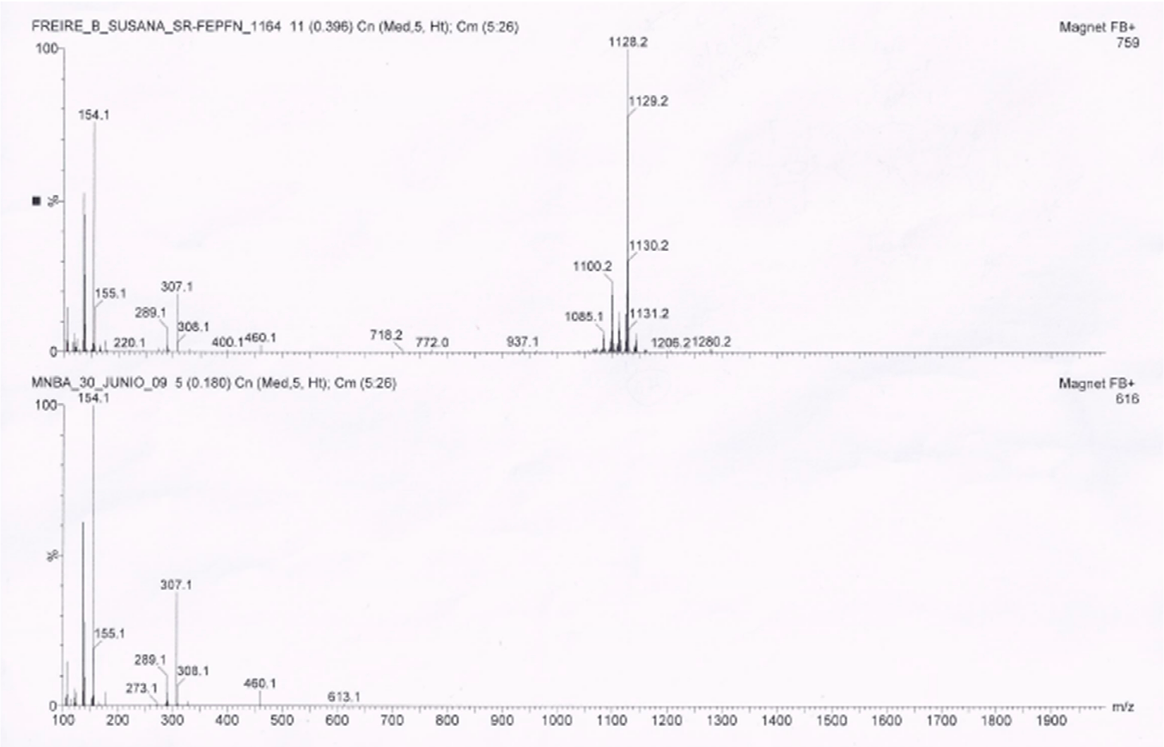

Figure S2. FAB[+] mass spectrum of Compound **V** [Fe(TF_4NMe_2PP)Cl] resulting from the reaction in Entry 1, Table 1 and comparison with the MNBA reference.

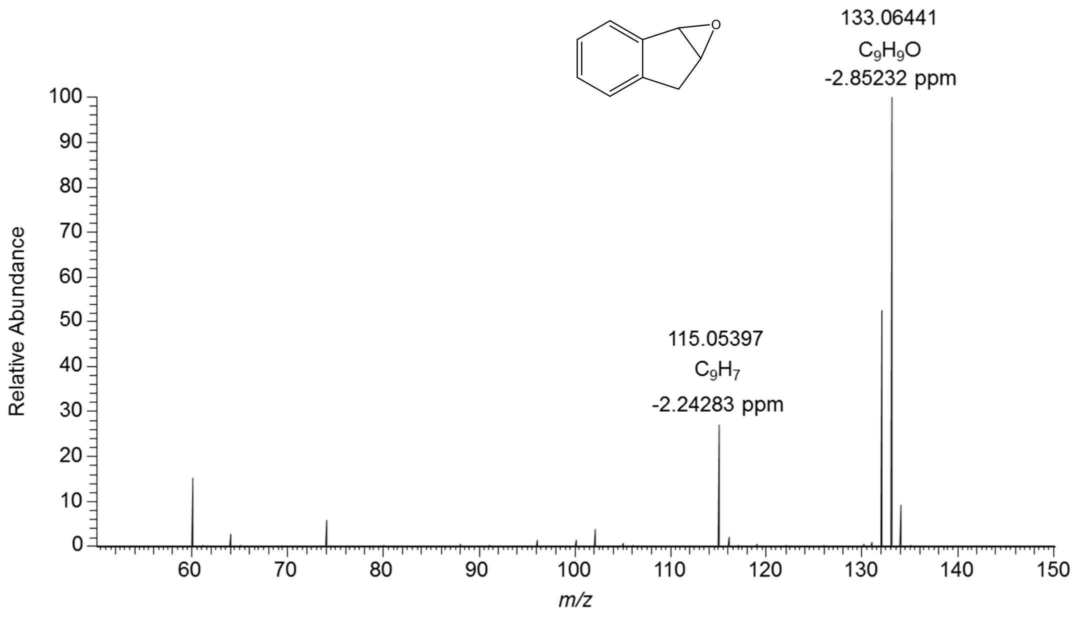

Figure S3. High resolution ESI+ mass spectrum of the final reaction mixture of compound **1** oxidation.

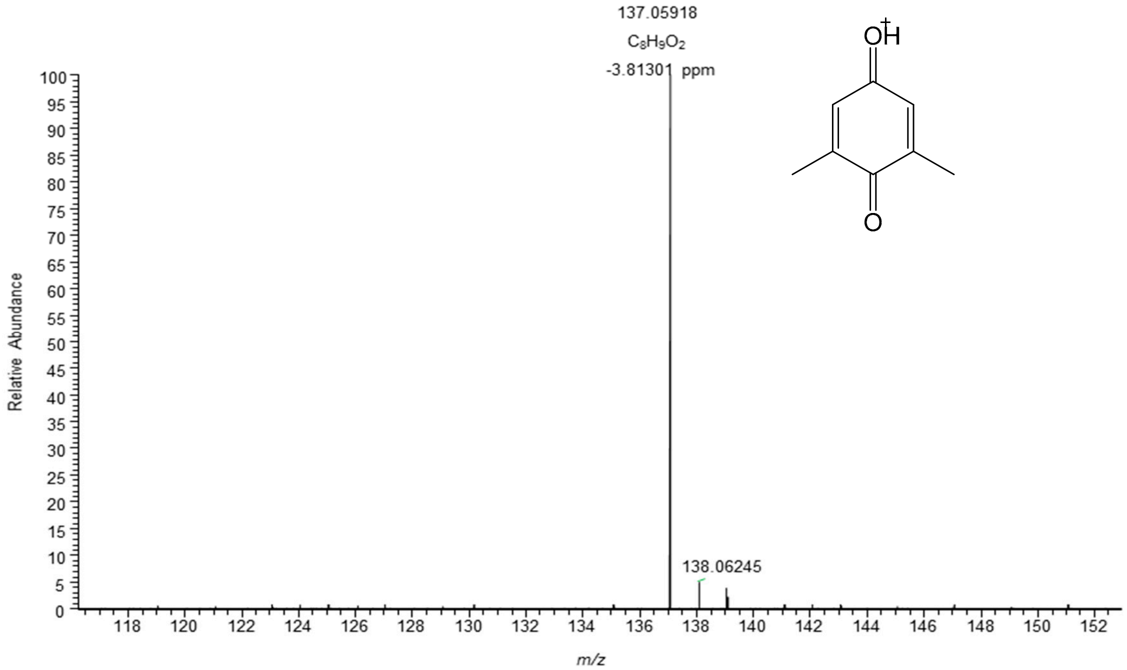

Figure S4. High resolution ESI+ mass spectrum of the final reaction mixture of compound **3** oxidation.

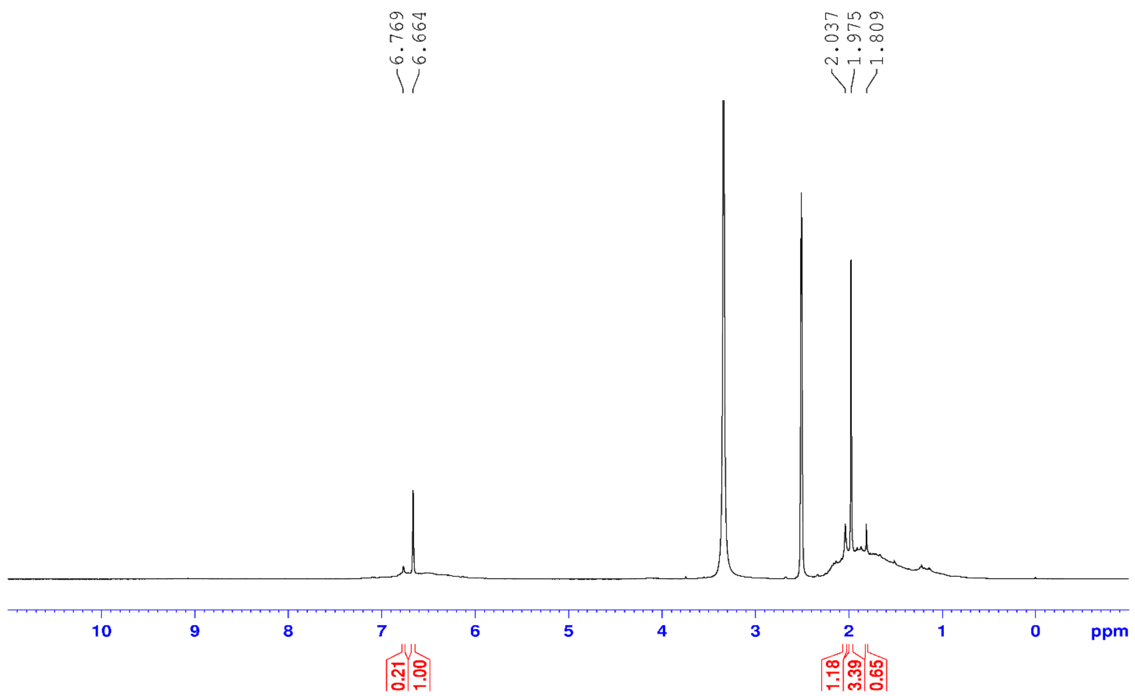

Figure S5. ^{1}H-NMR spectrum (in DMSO-*d*6) of the total reaction mixture present at the end of oxidation of compound **3**.

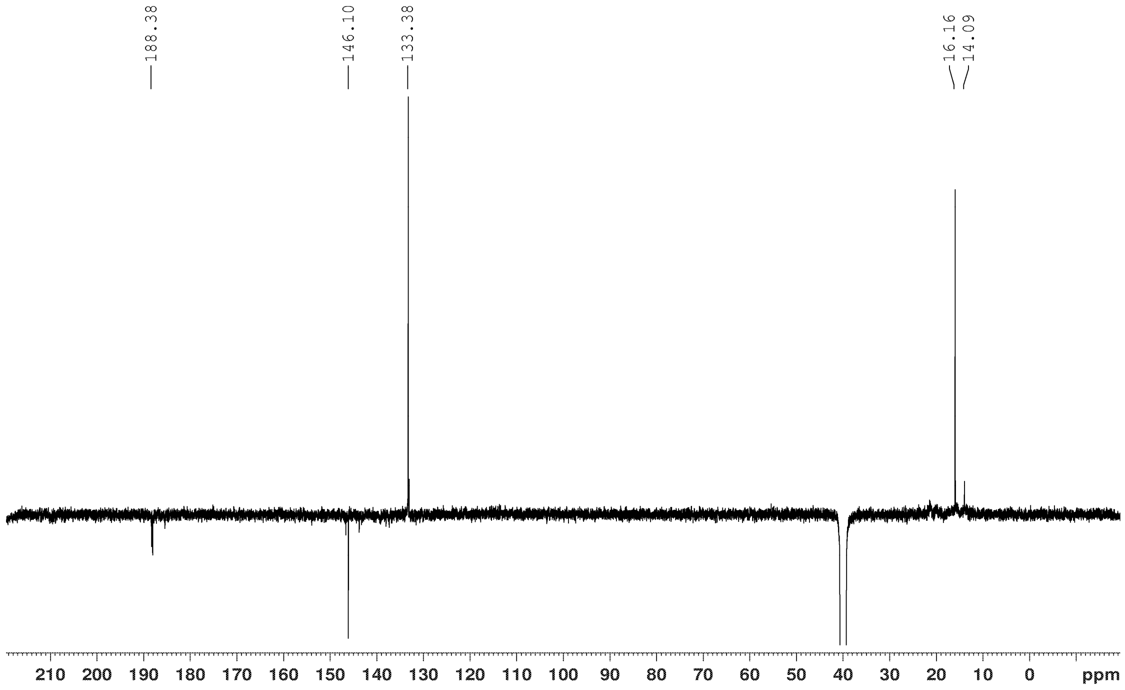

Figure S6. APT-NMR spectrum (in DMSO-d6) of the total reaction mixture present at the end of oxidation of compound **3**.

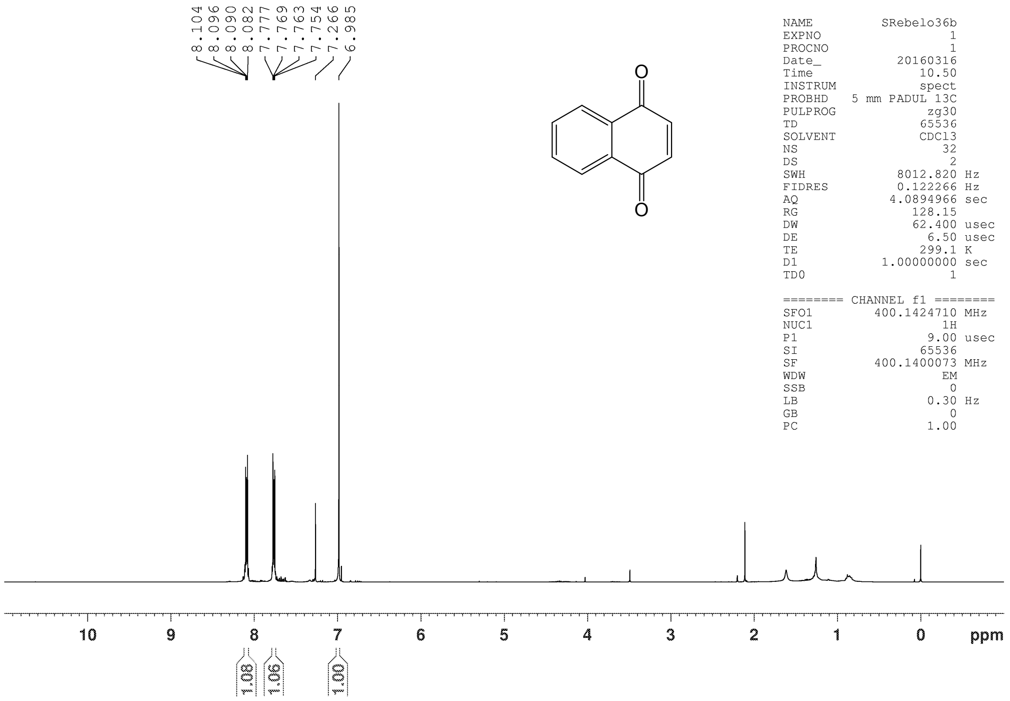

Figure S7. ^{1}H-NMR spectrum (in $CDCl_3$) of the total reaction mixture present at the end of the catalytic oxidation reaction of naphthalene **5**.

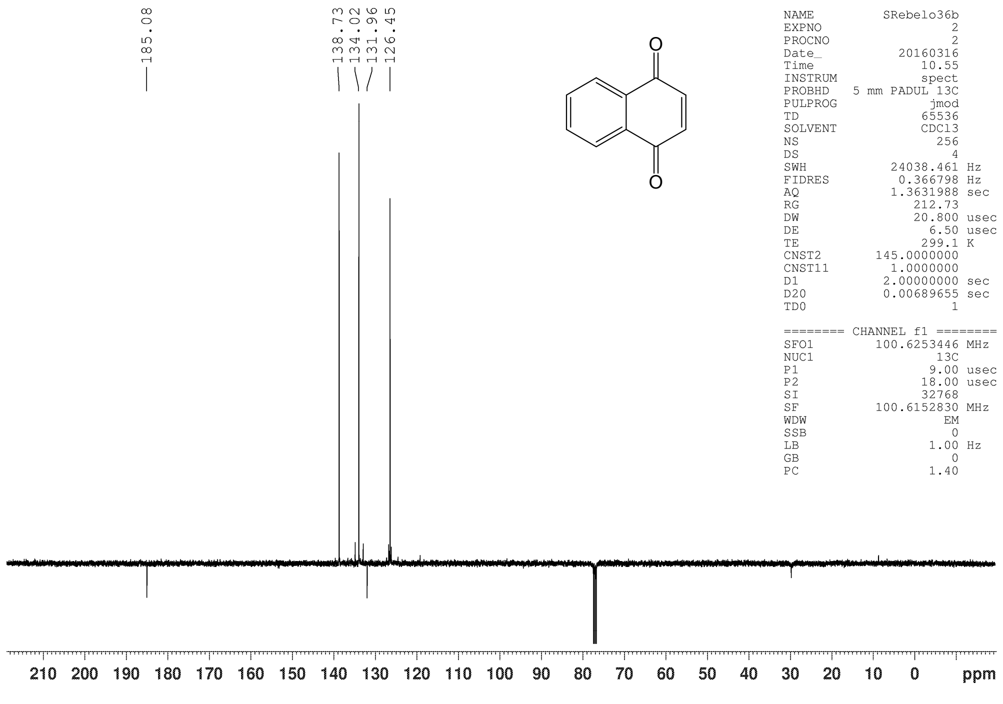

Figure S8. APT-NMR spectrum (in $CDCl_3$) of the total reaction mixture present at the end of the catalytic oxidation reaction of naphthalene **5**.

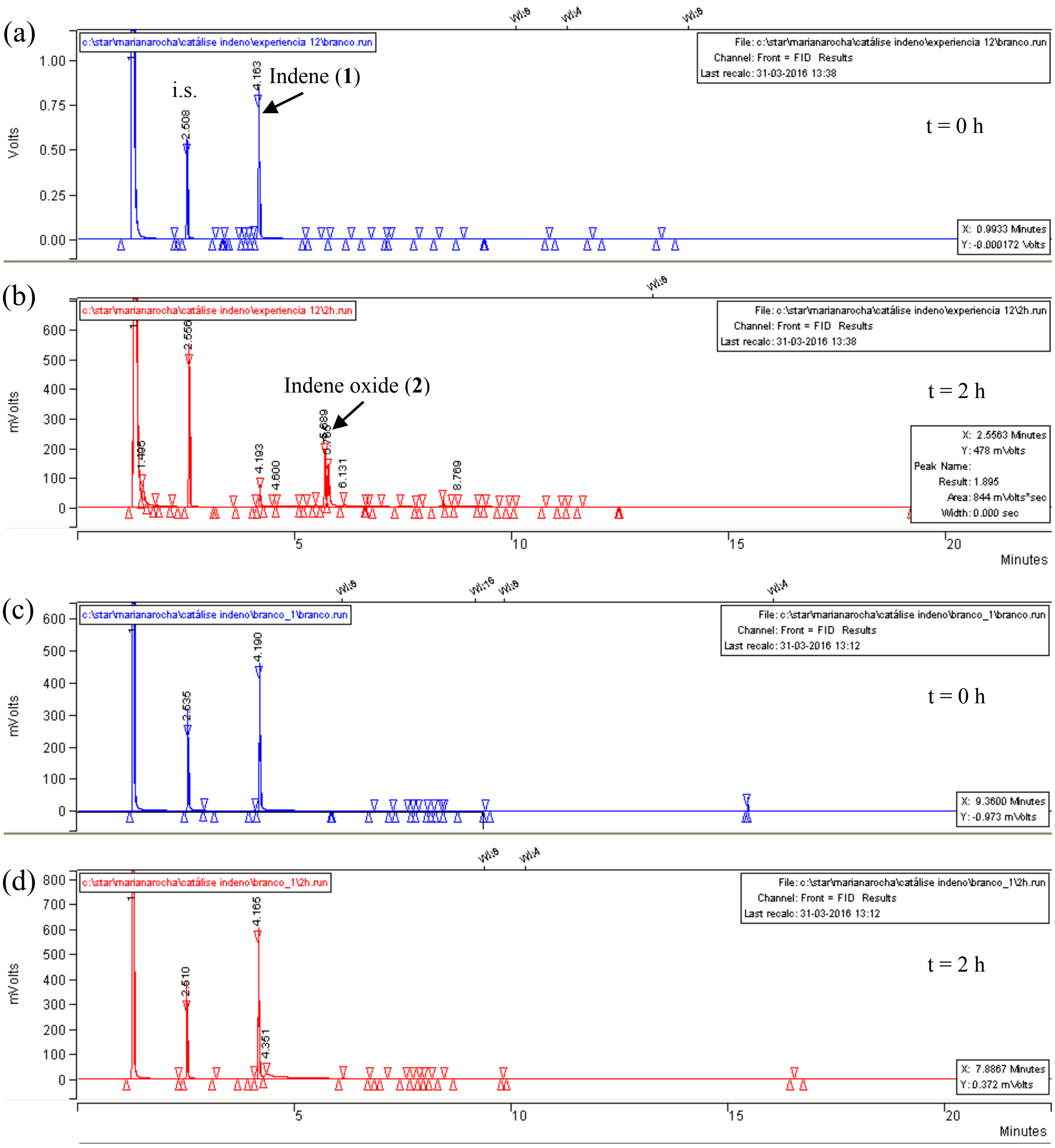

Figure S9. GC chromatograms of indene (**1**) oxidation at t = 0 h and t = 2 h of reactions in the presence (**a** and **b**) and absence (**c** and **d**) of catalyst. The relevant peaks are the internal standard (i.s.) chlorobenzene observed at retention time (r.t.) ~ 2.5 min; indene (**1**) r.t. ~ 4.2 min and indene oxide (2) (r.t. ~ 5.7 min) observed as two peaks due to the presence of two enantiomeric centers in the molecule.
